# Genetically engineered cell membrane-coated nanoparticles for antibacterial and immunoregulatory dual-function treatment of ligature-induced periodontitis

**DOI:** 10.3389/fbioe.2023.1113367

**Published:** 2023-01-25

**Authors:** Yangjia Deng, Mingxing Ren, Ping He, Fengyi Liu, Xu Wang, Chongjing Zhou, Yuzhou Li, Sheng Yang

**Affiliations:** ^1^ College of Stomatology, Chongqing Medical University, Chongqing, China; ^2^ Chongqing Key Laboratory of Oral Diseases and Biomedical Sciences, Chongqing, China; ^3^ Chongqing Municipal Key Laboratory of Oral Biomedical Engineering of Higher Education, Chongqing, China

**Keywords:** cell membrane coating, biomimetic nanoparticles, periodontitis, antibacterial therapy, immune regulation, TLR4

## Abstract

**Purpose:** In order to overcome the problem that conventional pharmacological treatments of periodontitis cannot effectively synergizing antimicrobial and immunomodulation, inspired by the critical role of toll-like receptor 4 (TLR4) in bacterial recognition and immune activation, we demonstrated a combined antibacterial-immunoregulatory strategy based on biomimetic nanoparticles.

**Methods:** Functioned cell membranes and silk fibroin nanoparticles (SNs) loaded with minocycline hydrochloride (Mino) were used to prepare a biomimetic nanoparticle (MSNCs). SNs and MSNCs were characterized by Scanning Electron Microscope, size, zeta potential, dispersion index. At the same time, SNs were characterized by cell counting kit-8 and real-time Polymerase Chain Reaction (RT-PCR). TLR4-expressing cell membranes were characterized by RT-PCR and western blot (WB). Cell membrane coating was characterized by Transmission Electron Microscope (TEM), the Bradford staining and WB. Then, Laser confocal, flow cytometry and agar plate coating were evaluated in vitro with antibacterial effects, RT-PCR was simultaneously evaluated with immunoregulatory effects. Finally, Anti-inflammatory treatment of MSNCs was evaluated in a ligature-induced periodontitis (LIP) mouse model.

**Results:** Successfully prepared cell membranes overexpressing TLR4 and constructed MSNCs. *In vitro* studies had shown that MSNCs effectively targeted bacteria via TLR4 and acted as molecular decoys to competitively neutralize lipopolysaccharide (LPS) in the microenvironment as well as inhibit inflammatory activation of macrophages. *In vivo*, MSNCs effectively attenuated periodontal tissue inflammation and alveolar bone loss in a LIP mouse model.

**Conclusion:** MSNCs have good targeted antibacterial and immunoregulatory effects, and provide a new and effective strategy for the treatment of periodontitis and have good potential for application in various types of pathogenic bacterial infections.

## 1 Introduction

The high prevalence of periodontitis is not only a major cause of tooth loss, but also a potential risk factor for diseases such as rheumatoid arthritis, cardiovascular disease and gastric cancer (Kinane et al., 2017; Hoare et al., 2019; Chen et al., 2021). The pathological features of periodontitis are mainly characterized by bacterial infection and local immune disorders, therefore antimicrobial therapy and regional immunomodulation are important tools in the treatment of periodontitis (Teng et al., 2000). However, the conventional mechanical treatment-based modalities still suffer from the problem of easy reattachment of plaque after treatment, which affects the therapeutic effectiveness (Slot et al., 2020). Therefore, basic treatment followed by adjuvant pharmacotherapy is increasingly considered to be the key to better treatment of periodontitis. Nevertheless, the current pharmacological treatment regimens for periodontitis still suffer from incomplete elimination of pathogenic bacteria and lack of improvement in immune dysfunction (Szulc et al., 2018). Consequently, the development of therapeutic regimens with both antibacterial and local immunoregulatory functions for the specific pathological microenvironment of periodontitis would help to solve the current dilemma.

It is well known that innate immunity, as the first line of defense against bacterial invasion, relies heavily on pattern recognition receptors (PRRs) (Zhou et al., 2018). In particular, recognition of pathogenic bacteria by neutrophils, macrophages and dendritic cells through toll-like receptors (TLR) is a key pathway for activating innate immunity (Federico et al., 2020). Numerous studies have found that LPS is one of the most important components of bacterial immune stimulation in periodontitis and is a key factor in periodontitis triggering systemic inflammation and sepsis (Rathinam et al., 2019). TLR4, as the predominant bacterial endotoxin receptor that recognizes innate immunity, plays a vital role in the activation of the immune response in periodontitis (Gu and Han, 2020). Innate immune cells recognize LPS released by bacteria in the microenvironment through TLR4 and activate inflammatory signaling pathways. In addition, they can also recognize and capture bacteria through TLR4 binding to LPS on the bacterial surface (Tsukamoto et al., 2018; Wang et al., 2018). Therefore, TLR4 in immune activation wound provide innovative thinking for the treatment of periodontitis. However, synergistic TLR4-based therapeutic approaches for precise antibacterial and immunoregulatory remain to be developed.

Nanodelivery systems are an important means to achieve targeted drug delivery and local immunomodulation (Sahu et al., 2021). Targeted drug delivery using nanoparticles is usually functionally modified using cationic groups (Hu et al., 2017; Huang et al., 2017), peptides (Huang et al., 2017), antibodies (Lambert and Berkenblit, 2018), *etc.* Surface membrane biomimetic modification of nanoparticles is a novel and efficient way. The cell membrane is coated around the nanoparticle surface by physical extrusion, ultrasonic or electroporation methods, which gives the nanoparticles bionic properties and preserves the protein function of the source cell membrane (Ai et al., 2020; Zou et al., 2020). For example, erythrocyte membranes (Zhang L. et al., 2018), leukocyte membranes (Parodi et al., 2013), platelet membranes (Li et al., 2018), and bacterial membranes (Gao et al., 2015) have been successfully used to prepare membrane-coated nanoparticles and provide an effective targeting delivery platform for antimicrobial and antitumor therapies. Additionally, the innovative applications of genetic engineering technologies can overexpress or newly express desired target proteins on cell membrane, highlighting and amplifying the functions of the key proteins (Ai et al., 2020), which also provides more expansion possibilities for the application of cell membrane bionanotechnology in targeted drug delivery and immune microenvironment modulation.

**FIGURE 1:**
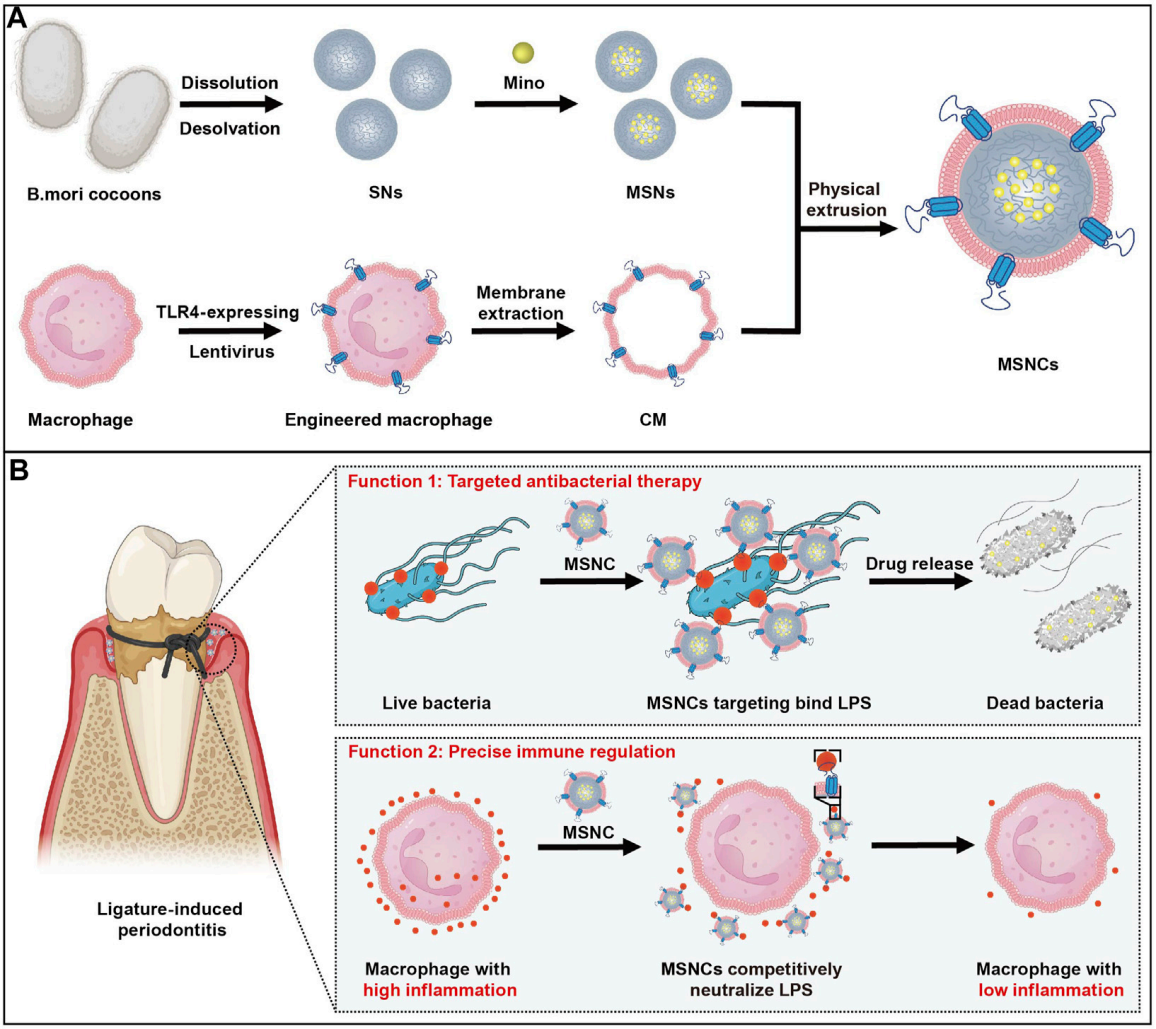
The fabrication and application of functioned cell membrane coated silk fibroin nanoparticles loaded with minocycline hydrochloride. **(A)** MSNCs are fabricated by coating engineered macrophage membranes onto SNs. **(B)** Application of MSNCs with targeted antimicrobial and precise immune regulation through topical administration of periodontitis.

Herein, inspired by the critical role of TLR4 in the recognition of pathogenic bacteria invasion and activation of inflammatory responses, we have innovatively developed a TLR4-expressing nanoplatform with cell membrane bionic effects. The nanoplatform was genetically engineered to construct TLR4-expressing RAW264.7 macrophages and extract their cell membranes, and then the functionalized cell membranes were physically extruded to coat the surface of SNs loaded with the antimicrobial agent MINO to prepare nanodelivery systems MSNCs. With this novel dual-functional construction strategy, nanoparticles with both targeted bactericidal and immunomodulatory functions were successfully synthesized. *In vitro* experiments demonstrated that MSNCs had good targeting to *Escherichia coli* (*E. coli*) and can release antibacterial drugs to kill bacteria efficiently. Moreover, they effectively inhibited the inflammatory activation of macrophages by neutralizing LPS in the microenvironment. *In vivo* studies showed that MSNCs effectively suppressed periodontal inflammation and reduced alveolar bone resorption in a ligature-induced periodontitis (LIP) mouse model (Figure 1).

## 2 Materials and methods

### 2.1 Reagents and materials

*Bombyx mori* (B.mori) cocoons were donated from Southwest University (Chongqing, China); TLR4 lentivirus was purchased from Genechem Co. (Shanghai, China); FITC and LPS were purchased from Sigma-Aldrich (St. Louis, MO, United States); Cell counting kit-8 was purchased from MCE (New Jersey, United States); Sodium carbonate, lithium bromide, paraformaldehyde and hematoxylin, and eosin staining kit were purchased from Solarbio Science and Technology Co. (Beijing, China); acetone was purchased from CHUANDONG CHEMICAL (Chongqing, China); Membrane and Cytosol Protein Extraction Kit, Dil, BCA Protein Assay Kit and DAPI were purchased from Beyotime Biotechnology (Shanghai, China).

### 2.2 Cell and bacterial culture

RAW264.7 cells is a macrophage cell line (American Type Culture Collection, ATCC) that was established from a tumor in a male mouse induced with the Abelson murine leukemia virus. And the cell line was used for macrophage membranes collection in the current study. RAW264.7 cells cultured in Dulbecco’s modified Eagle’s medium (H-DMEM, Sigma) supplemented with 10% fetal bovine serum (FBS, Excell Bio) and 1% penicillin-streptomycin (HyClone, United States) at 37°C in a 5% CO_2_ atmosphere. Gram-negative bacteria *E. coli* (ATCC 43888) was grown in LB medium at 37°C in an aerobic environment, and bacteria used in all experiments were in the mid-exponential growth period. The bacterial suspension was diluted to 1*10^6^ colony forming units (CFU per mL) for experimental use after measuring the optical density at 600 nm using a microplate reader (ELX800, Gene Company Limited, China).

### 2.3 Preparation and characterization of SNs

Silk fibroin solution was prepared as previously described (Xiang et al., 2022). Briefly, B.mori cocoons were cut into pieces and then boiled in 0.02 M sodium carbonate for 60 min and then completely rinsed and dried in the convection oven at 37°C for 24 h. Next, the degummed silk fibroin was dissolved using 9.3 M lithium bromide at 60°C for 4 h and dialyzed in double-distilled water for 72 h. Then, the silk fibroin solution was purified by centrifugation at 5,000 rpm. As reported in the literature (Wongpinyochit et al., 2016), 5% silk fibroin solution was added dropwise to acetone, and nanoparticles were precipitated using a high-speed centrifuge at 32,000 g for 2 h at 4°C. The supernatant was removed and resuspended in double-distilled water, the nanoparticles were dispersed by vortex and ultrasound probe, and repeated twice. Finally, the nanoparticles were resuspended using double-distilled water and stored at 4°C until used. The morphology of SNs was observed by scanning electron microscopy (Hitachi SU8010, Japan). Mean particle size and potential were detected by a dynamic light scattering (DLS) detector (Brookhaven NanoBrook Omni, United States). Cytotoxicity assay was verified by cell counting kit-8. Biocompatibility was confirmed by real-time polymerase chain reaction (Bio-Rad, United States).

### 2.4 Mino loading assay of SNs

Add the corresponding mass of Mino in SNs suspension at different mass ratios (Mino: SNs = 0.125, 0.375, 0.5) and stir away from light overnight at room temperature. The supernatant was centrifuged at 32,000 g for 30 min, and the double-distilled water was resuspended and repeatedly washed twice before being stored at 4°C, protected from light. The supernatant was collected and used to calculate the encapsulation efficiency and drug loading efficiency. The equations of drug loading and encapsulation efficiency were described as follows:

$$Drug\;loading\;efficiency=\frac{Weight\;of\;feeded\;drug-Drug\;amount\;in\;supernatant}{Weight\;of\;dry\;NPs}\times 100\;\%$$

$$Encapsulation\;efficiency=\frac{Weight\;of\;feeded\;drug-Drug\;amount\;in\;supernatant}{Weight\;of\;feeded\;drug}\times 100\;\%$$

### 2.5 Genetic engineering of RAW264.7

Lentiviral transfection of RAW264.7 cells was performed according to the product instructions. Briefly, RAW264.7 cells (50 000/well) were incubated for 12 h, then appropriate H-DMEM, TLR4 lentivirus (MOI = 50), and empty lentivirus were added separately to the culture medium for 10 h. After 48 h of incubation, the cells were harvested for the TLR4 mRNA levels by RT-PCR. Among them, the medium-treated group served as the control group for the experiment, the blank vector lentivirus group served as the negative control (NC) group, and the TLR4 lentivirus-treated group served as the TLR4 group. The sequences of the primers (Takara, Japan) were shown in Table 1. Cells were selected by adding 4 μg/mL puromycin in serum-containing medium for 2 days after passaging and continued to transfect the cell line with 2 μg/mL puromycin to maintain steadily for 1 w. An antibody against TLR4 (sc-293072, Santa) was used to detect markers in cell homogenization by Western blot.

**TABLE 1:** The prime sequences of all genes used in RT-PCR.

Gene	Forward primer (5′–3′)	Reverse primer (5′–3′)
TLR4	TTC​ACC​TCT​GCC​TTC​ACT​ACA	GGG​ACT​TCT​CAA​CCT​TCT​CAA
IL-1β	TTG​AAG​TTG​ACG​GAC​CCC​A	GAG​TGA​TAC​TGC​CTG​CCT​GAA​G
IL-6	GTT​GCC​TTC​TTG​GGA​CTG​ATG	TTG​GGA​GTG​GTA​TCC​TCT​GTG​A
GAPDH	GCA​CCG​TCA​AGG​CTG​AGA​AC	TGG​TGA​AGA​CGC​CAG​TGG​A

### 2.6 Preparation and characterization of SNCs

CMs extraction was performed according to Membrane and Cytosol Protein Extraction Kit. Briefly, functionalized macrophages were repeatedly rinsed with PBS after collection and cell counting. The cells were resuspended with Membrane Protein Extraction Reagent A containing PMSF. After 10 min of an ice bath, the cell suspension was homogenized using an ultrasonic disruptor and centrifugated at 700 g for 10 min, then the supernatant was collected and centrifugated at 14,000 g for 30 min, and resuspended the precipitate with PBS. Eventually, the concentration of CMs suspension was determined by the BCA method, and the suspension was stored at −80°C for use. The extracted CMs and SNs were mixed in a 1:1 mass ratio and repeatedly extruded through a polycarbonate membrane with the aid of a liposome extruder to obtain MSNCs. The morphology and structure of MSNCs were separately observed by scanning electron microscopy and transmission electron microscopy (JEOL JEM-1400PLUS, Japan). Mean particle size and potential were detected by DLS. Finally, the Bradford method and WB were used to verify the successful coating of the CMs. After preparation of MSNCs, two equal groups of MSNCs were added into dialysis bags with appropriate amount of *E. coli* in one group. The dialysis bags were completely submerged in PBS (PH = 7.4) and drug release was monitored at a constant temperature of 37°C for 0, 1, 4, 8, 12, 24, and 48 h.

### 2.7 Targeting binding of MSNCs to bacteria *in vitro*

The red fluorescent dye Dil and the green fluorescent dye FITC were used to label CMs and SNs, respectively. Dil -SNCs were prepared by a liposome extruder after mixing Dil with CMs for 2 h at room temperature; FITC-SNs were prepared by mixing SNs with FITC overnight away from light and purified by dialysis. *E. coli* suspension was fixed with paraformaldehyde and washed twice with PBS. Dil-SNCs were added to the experimental group, incubated at 37°C for 1 h, stained with DAPI for 3 min, and resuspended after centrifugation to obtain the suspension. The suspension was dropped on slides for sample preparation and then images were acquired by laser confocal microscopy (Leica TCS SP8, Germany). Meanwhile, after paraformaldehyde fixation of *E. coli*, the experimental groups were incubated with FITC-SNs and FITC-SNC individually for 1 h at 37°C and washed for flow cytometry detection (BD Bioscience, United States).

### 2.8 Antibacterial effects of MSNCs

*E. coli* were inoculated on 24-well plates, and SNCs and MSNCs were added to the experimental groups, respectively, and incubated for 24 h in a 37°C bacterial incubator. The incubated mixture was serially diluted and coated on nutrient agar plates, incubated for 24 h in a 37°C bacterial incubator, and photographed to observe the growth of colonies in the plates.

### 2.9 LPS neutralization of SNCs *in vitro*

RAW264.7 cells were used to detect SNCs biocompatibility and LPS neutralization. Cells were inoculated with a complete Medium in the control group and 40, 80, 120, 160, and 200 μg/mL of SNCs H-DMEM medium suspension in the experimental group, and cell viability was detected using the CCK-8 kit after 24 h of incubation. In addition, the experimental groups were incubated with LPS, LPS + SNs, and LPS + SNCs for 12 h, after being incubated in the cell incubator for 24 h. RT-PCR was performed as before.

### 2.10 The biological role of MSNCs *in vivo*

6-week-old male C57 mice were provided by the laboratory Animal Center of Chongqing Medical University. Periodontitis molds were completed by ligating the cervical portions of the bilateral maxillary first and second M of C57 mice for 1 week using 5–0 silk wire. To study the diffusion of MSNCs at the site of inflammation, we prepared FITC-labeled MSNCs and injected FITC-MSNCs into the palatal gingiva of mice with ligature-induced periodontitis, collected at 0, 4, and 24 h, and observed their diffusion in the periodontium by fluorescence distribution under a stereomicroscope. Then, all mice were divided into four groups, no treatment in the control group, no treatment in the periodontitis group after completion of periodontal ligation, and the rest of the experimental groups were administered with the effective drug concentration of 0.5 mg/ml in MSNs and MSNCs suspension respectively at the ligated tooth position by drops of 5 μl on each side for 1 week. Mice were anesthetized and executed after 1 week, and periodontal tissues were taken from bilateral ligated tooth sites for RT-PCR, WB, and Hematoxylin Eosin (HE) staining to detect the inflammatory changes. An antibody against IL-1β (ab9722, Abcam) was used to detect markers in cell homogenization by WB. Animal handling and surgical procedures were performed following the protocol approved by the Ethics Committee of the Affiliated Stomatological Hospital of Chongqing Medical University (2022 (No. 062)).

### 2.11 Statistical analysis

All experimental data are presented as the mean ± standard error of the mean (SEM), and the results were analyzed using a one-way analysis of variance (ANOVA) among groups followed by Tukey’s *post hoc* test. A value of *p <* 0.05 was considered statistically significant (*p < 0.05, **p < 0.01, ***p < 0.001, ****p < 0.0001).

## 3 Results

### 3.1 Preparation and characterization of SNs

**FIGURE 2:**
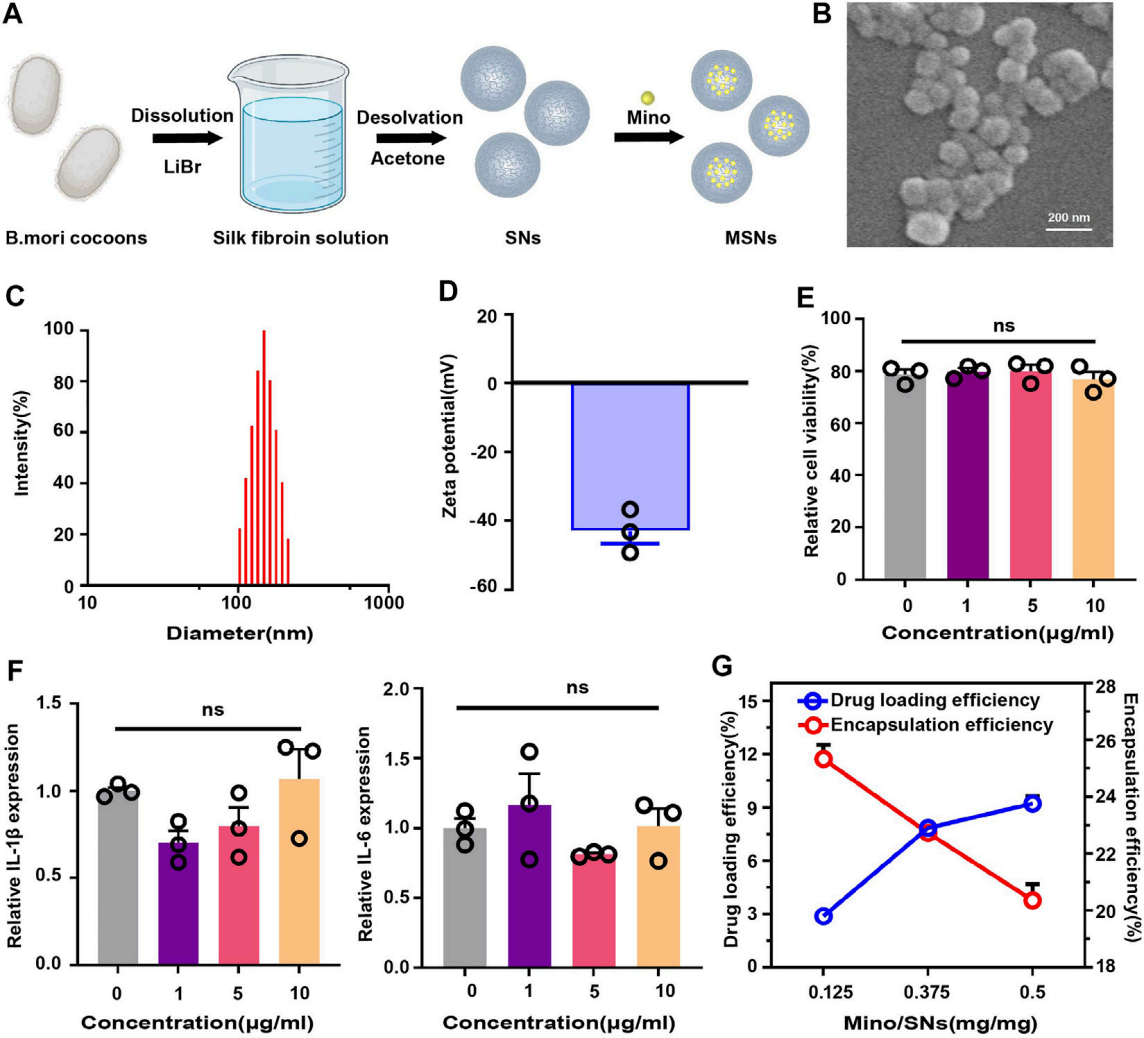
Characterization of silk fibroin nanoparticles. **(A)** Schematic illustration of the synthesis of drug-loaded SNs. **(B)** Scanning electron microscopy of SNs. **(C)** Particle size distribution of SNs. **(D)** Zeta potential diagram of SNs. **(E)** CCK-8 of SNs. **(F)** RT-PCR of SNs at different concentrations. **(G)** Drug loading and encapsulation efficiency for different mass ratios of Mino and SNs. Data are presented as means ± SEM, ns *p* > 0.5; by one-way analysis of variance (ANOVA) with Tukey’s *post hoc* test **(E** and **F)**.

SNs were prepared by the desolvation method and loaded with Mino (Figure 2A). Scanning electron microscopy showed that the nanoparticles were spherical together with a clear boundary, and the diameter was about 150 nm (Figure 2B). DLS showed that the size of SNs was 114.63 ± 4.55 nm (Figure 2C), zeta surface potential was −39.70 ± 2.77 mV (Figure 2D), and polydispersity index (PDI) was 0.31 ± 0.02. The CCK-8 assay indicated that the viability of RAW264.7 cells had no significant difference (*p* > 0.05) to the increased concentration of SNs (Figure 2E). The gene expression of both IL-1β and IL-6 in RAW264.7 cells was of no meaningful difference (*p* > 0.05) to the increased concentration of SNs (Figure 2F). There different mass ratios of Mino: SNs (Mino: SNs = 0.125, 0.375, 0.5) were examined, and the drug loading efficiency and encapsulation efficiency of Mino were determined, as shown in Figure 2G. When the ratio increased from 0.125 to 0.375, the drug loading efficiency of Mino increased from 2.89% to 7.86%. However, when the ratio increased to 0.5, the drug loading efficiency increased slightly to 9.24%. And the encapsulation decreased all the time from 25.34% to 20.36%. Therefore, Mino and SNs with a mass ratio of 0.375 were chosen for the follow-up experiment owing to their most suitable drug loading rate and encapsulation rate.

### 3.2 Preparation and characterization of MSNCs

**FIGURE 3:**
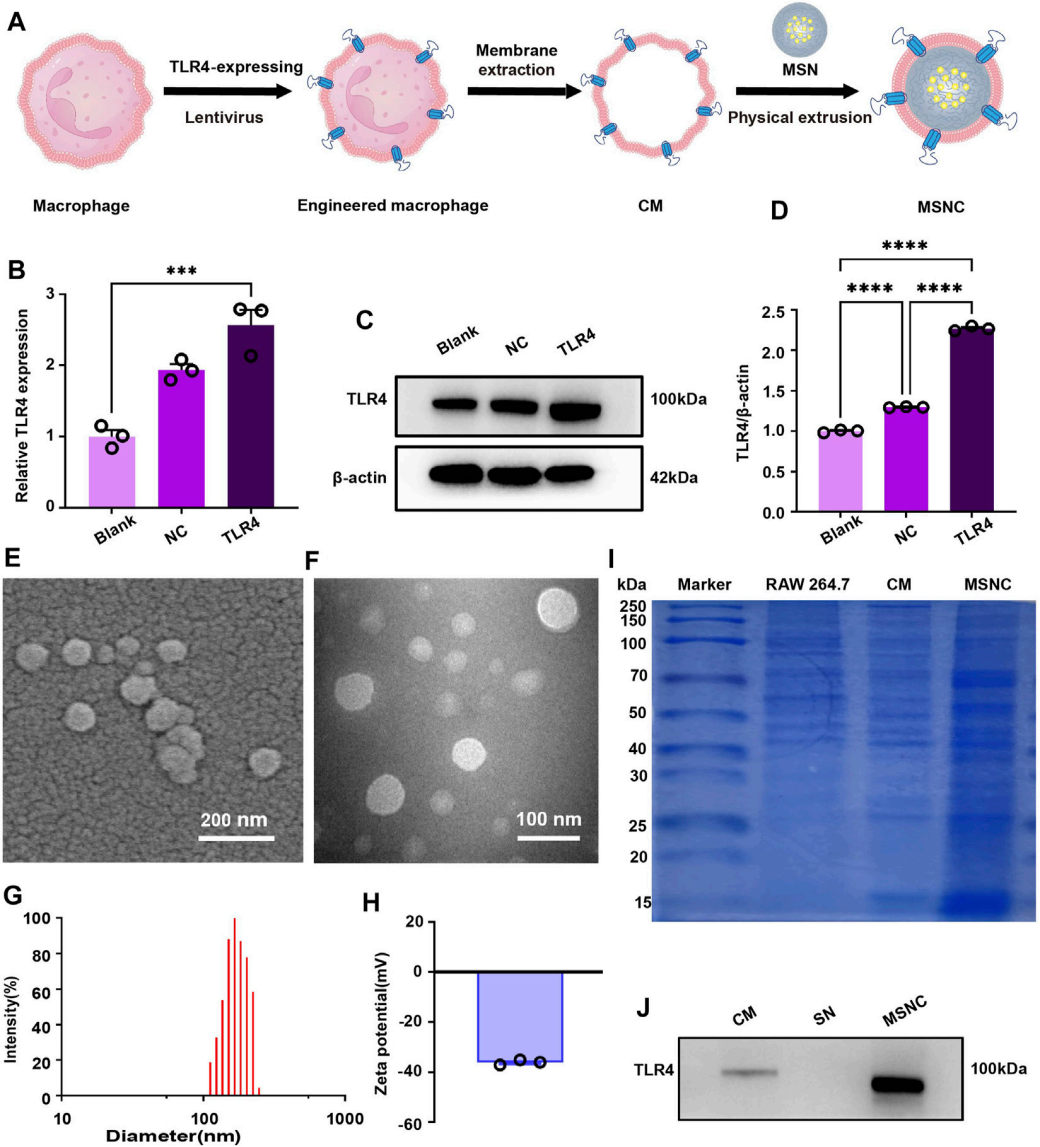
Characterization of functioned cell membrane coated silk fibroin nanoparticles loaded with minocycline hydrochloride. **(A)** Schematic illustration of MSNCs. **(B)** TLR4-expressing in RAW264.7 cells by RT-PCR. **(C)** TLR4-expressing in RAW264.7 cells by WB. **(D)** Quantitative analysis of the WB. **(E)** Scanning electron microscopy of MSNCs. **(F)** TEM of MSNCs. **(G)** Particle size distribution of MSNCs. **(H)** Zeta potential diagram of MSNCs. **(I)** The protein composition of MSNCs was verified by SDS-PAGE followed by the Bradford staining. **(J)** The successful translocation of CMs proteins was verified by WB. Data are presented as means ± SEM, ***p < 0.001, and ****p < 0.0001; by one-way analysis of variance (ANOVA) with Tukey’s *post hoc* test **(B,D)**.

TLR4-expressing RAW264.7 cells were constructed by lentivirus, RT-PCR, and WB assays showed that the TLR4 gene and protein expression had increased in TLR4 treantment, compared control group (Figures 3B–D). After the preparation of MSNCs (Figure 3A), scanning electron microscopy showed that the surface of MSNCs was covered with an unsmooth film structure (Figure 3E), and TEM showed that MSNCs had a ring-like structure, the density of the ring layer was the highest, the density inside the ring was the second highest, and the density outside the ring was the lowest (Figure 3F). The DLS results showed that the MSNCs size was 134.04 ± 1.50 nm (Figure 3G), the zeta potential was −36.09 ± 0.98 mV, and PDI was 0.37 ± 0.98 (Figure 3H). Therefore, the size of MSNCs increased by about 20 nm compared with the size of SNs. Above all, the results of DLS and TEM tentatively proved the successful preparation of MSNCs. As shown in Supplementary Figure S1, the combination with bacteria does not affect the continuous release of the drug and helps to slow down the initial slow release of the drug, and the later release of the drug is the same as usual.

### 3.3 Verification of cell membrane coat to MSNCs

The results of protein bands after Bradford staining showed that the three groups of protein bands of cell homogenate, CMs, and MSNCs were similar, indicating that the membrane components were similar, and the protein bands of MSNCs and CMs groups were highly overlapping (Figure 3I). It represented the successful coat of CMs on the surface of MSNCs. Moreover, TLR4 protein was present in both the CMs and MSNCs groups, but no TLR4 protein was expressed in the SNs group, indicating successful translocation of CMs proteins on the MSNCs surface (Figure 3J).

### 3.4 Targeting binding of SNCs to *E. coli*

**FIGURE 4:**
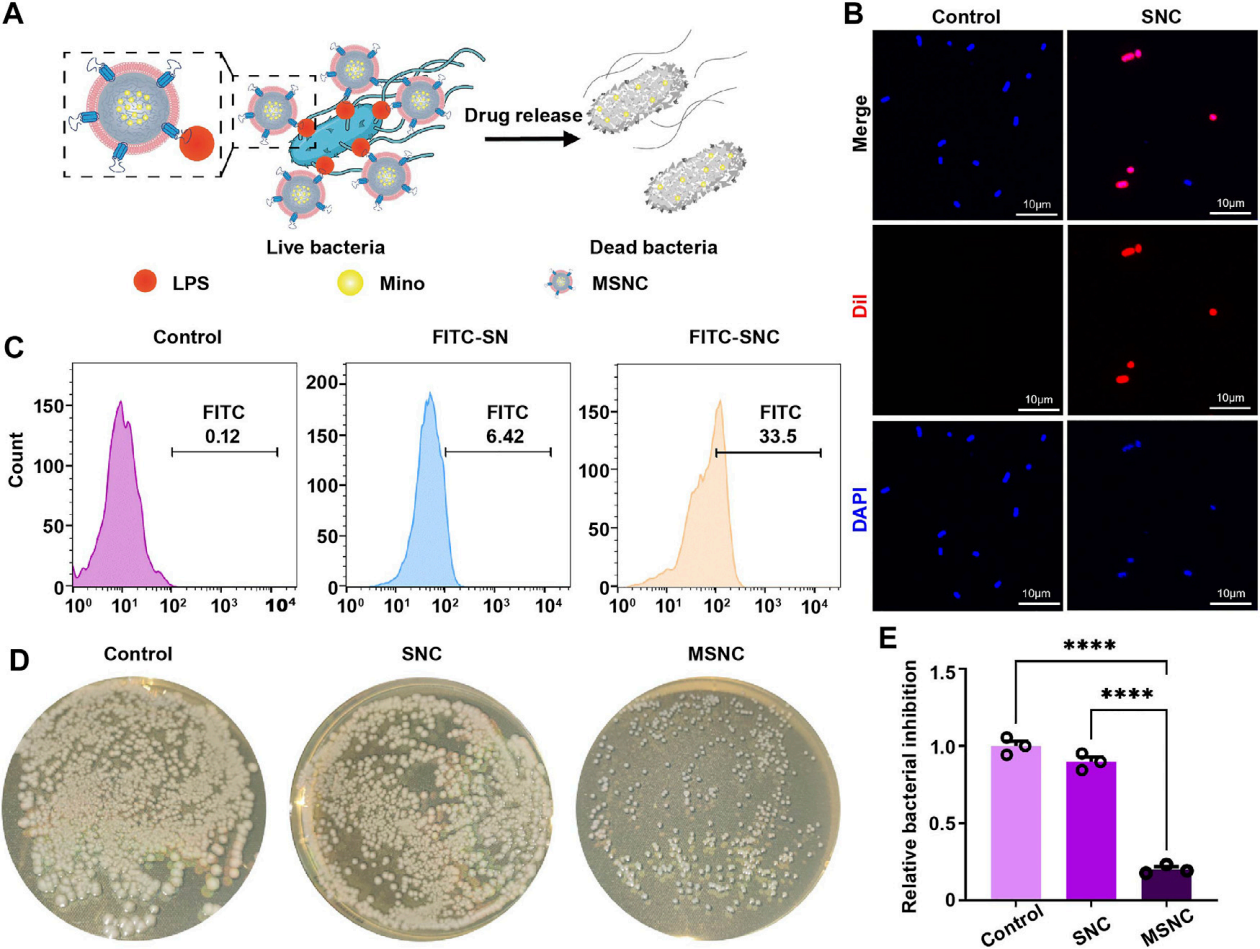
Targeting antibacterial effect of functioned cell membrane coated silk fibroin nanoparticles loaded with minocycline hydrochloride. **(A)** Schematic illustration of MSNCs targeting antibacterial effects. **(B)** Probing bacterial targeting using Dil-labeled cell membranes of SNCs with DAPI-labeled bacteria by laser confocal microscopy. **(C)** Probing bacterial targeting using FITC-labeling of SNCs by flow cytometry. **(D)** The antibacterial effect of MSNCs on *E. coli* in agar plates. **(E)** Quantitative analysis of the number of bacteria on agar plates. Data are presented as means ± SEM, ***p < 0.001, and ****p < 0.0001; by one-way analysis of variance (ANOVA) with Tukey’s *post hoc* test **(E)**.

To investigate the adherence of SNCs to *E. coli* (Figure 4A), Dil and FITC were used to mark CMs and SNs respectively, and then the adherence to *E. coli* was evaluated by laser scanning confocal microscopy and flow cytometry. Under the microscope, bacteria were marked in blue, CMs were marked in red. It would show a pink morphology when SNCs and *E. coli* co-localized (Figure 4B). In addition, the result of flow cytometry show that a low binding rate (6.42%) was found between SNs and *E. coli*. However, SNCs containing macrophage membranes presented a higher binding rate (33.5%), demonstrating that macrophage membrane camouflaging could improve the adherence to *E. coli* and achieve precise delivery of Mino (Figure 4C). In addition, the results of nutrient agar plate coating showed a good antibacterial effect on MSNCs, while the unloaded SNCs did not have an antibacterial effect (Figures 4D,E). It was suggested that Mino was successfully loaded into SNCs and effectively released within 24 h to exert antibacterial effects.

### 3.5 Immunoregulatory effects of SNCs

**FIGURE 5:**
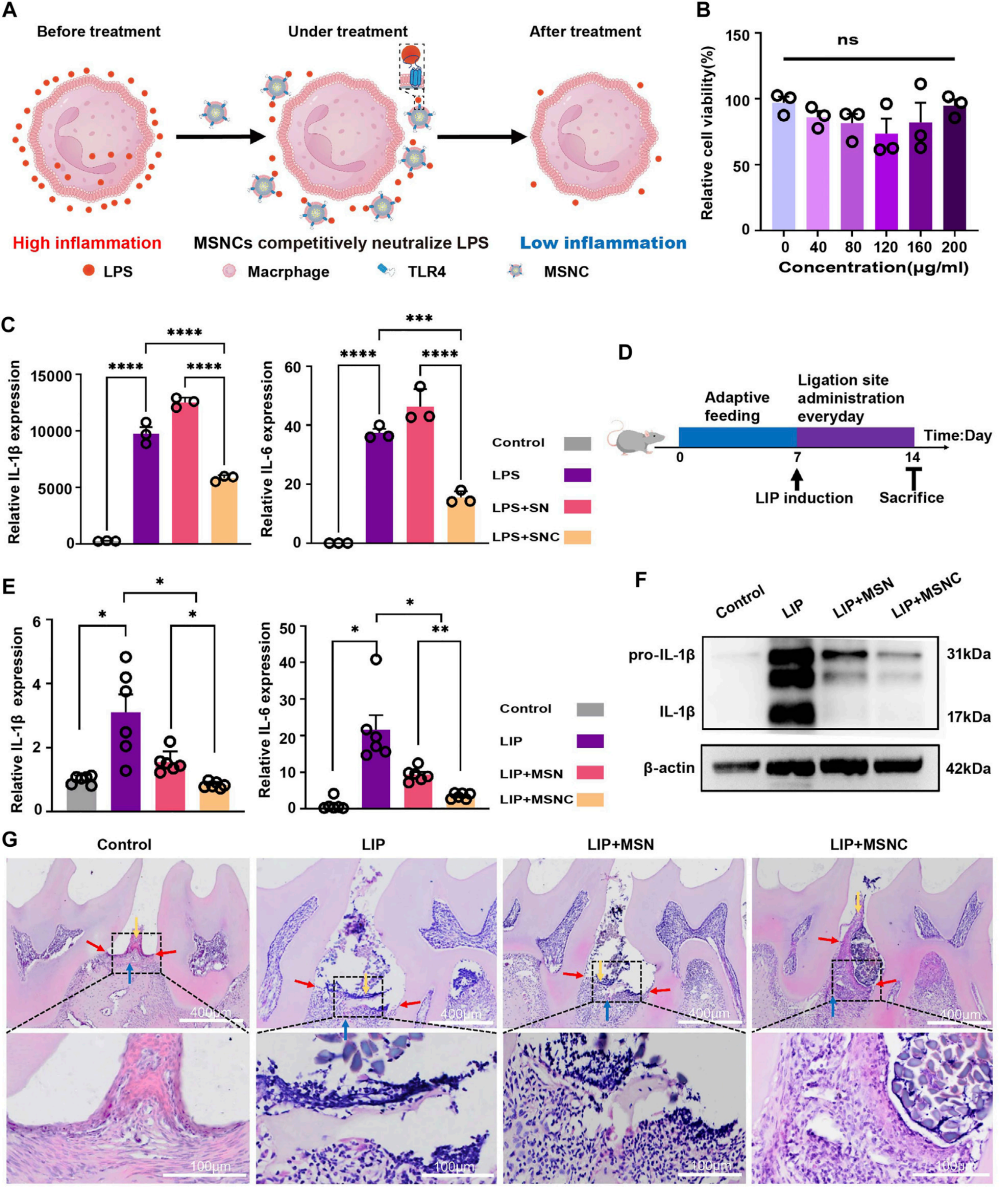
Anti-inflammatory effects of functioned cell membrane coated silk fibroin nanoparticles loaded with minocycline hydrochloride *in vitro* and vivo. **(A)** Schematic illustration of MSNCs immunoregulatory effects *in vitro*. **(B)** CCK-8 of SNCs. **(C)** RT-PCR for SNCs neutralization of LPS. **(D)** Schematic illustration of animal experiments. **(E)** RT-PCR of MSNCS with reducing periodontal tissue inflammation. **(F)** WB of MSNCs with reducing periodontal tissue inflammation. **(G)** HE staining of MSNCs with reducing periodontal tissue inflammation. The yellow arrow represents the position of the gingival epithelial peg, the red arrow represents the position of the junctional epithelium and the blue arrow represents the position of the alveolar bone. Data are presented as means ± SEM, ns *p* > 0.5, *p < 0.05, **p < 0.01, ***p < 0.001, and ****p < 0.0001; by one-way analysis of variance (ANOVA) with Tukey’s *post hoc* test **(B,C, E)**.

The TLR4 receptor on the surface of macrophage membranes might neutralize LPS and modulate the local immune microenvironment (Figure 5A). The CCK-8 assay indicated that the viability of RAW264.7 cells had no significant difference (*p* > 0.05) to the increased concentration of SNCs (Figure 5B). However, the gene expression of both IL-1β and IL-6 in RAW264.7 cells was markedly downregulated in SNCs groups (*p* < 0.001), indicating that SNCs competitively neutralized LPS in the microenvironment and effectively inhibited the activation of pro-inflammatory macrophages (Figure 5C).

### 3.6 Biological effects of MSNCs *in vivo*

A mouse model of ligature-induced periodontitis was constructed to evaluate the *in vivo* biological effects of MSNCs (Figure 5D). Significant diffusion was observed after FITC-MSNCs were injected into the tissue (Supplementary Figure S2). at 0 h, obvious green fluorescence was visible at the injection site with bright fluorescence and comparatively aggregated location; at 4 h, the fluorescence at the injection site attenuated and obvious green fluorescence diffusion was visible around; at 24h, the fluorescence at the injection site diminished significantly and the fluorescence range was larger, and obvious green fluorescence was visible at the periodontitis site. Moreover, RT-PCR assay showed that IL-1β and IL-6 gene expression had prominently decreased in the MSNCs group (*p* < 0.05), and WB assay suggested that IL-1β protein expression had significantly decreased in MSNCs (Figures 5E,F). Furthermore, HE staining showed that the gingival epithelial pegs proliferated (yellow arrows), the junctional epithelium receded toward the root (red arrows) and the alveolar bone height decreased (blue arrows) in the periodontitis group; Nevertheless, with the treatment of MSNCs, gingival epithelial peg proliferation combined with epithelial recession and alveolar bone loss improved significantly (Figure 5G).

## 4 Discussion

In this study, we constructed cell membrane-coated silk fibroin nanoparticles (MSNCs) overexpressing TLR4 by genetic engineering and proposed a new antibacterial and immunoregulatory synergistic therapy for periodontitis. The TLR4 was used to achieve precise targeted drug delivery and killing of pathogens, while nanoparticles with TLR4 acted as ideal decoys for LPS to reduce the activation of immune cells. The synergistic therapy based on MSNCs made up for the defects of the current treatment and realized the effective control of periodontal inflammation.

Nanoparticles are an effective platform to achieve precise drug delivery and cellular regulation (Wang et al., 2019; Mitchell et al., 2021). Nanoparticles can not only overcome the limitations of free drug therapeutic approaches, but also overcome the heterogeneity among different patients and diseases through targeted therapy (Tiwari et al., 2012; Wilczewska et al., 2012; Ren et al., 2021). In addition, the effects of nanoparticles on cellular viability and polarity have been widely researched to improve the disease microenvironment. Considering the effect of nanoparticles on cytotoxicity, natural polymers are often chosen as raw materials for the preparation of nanoparticles due to their good biocompatibility and degradability (Idrees et al., 2020; Wang et al., 2022; Yu et al., 2022). They are used for the pharmacotherapy of small molecule drugs, proteins, and nucleic acids, which are widely studied in the fields of tumor therapy, tissue engineering, and regenerative medicine (Balazs et al., 2006; Malafaya et al., 2007; Pina et al., 2015; Chen et al., 2019). In the present study, we prepared nanoparticles with a natural polymeric silk fibroin. The scanning electron microscopy results showed that the SNs had a circular morphology. The DLS results showed that the size and zeta potential were respectively about 114.63 nm and −39.70 mV, which were similar to the study by Shuang, Q. Gou (Gou et al., 2019). We demonstrated that SNs had no significant cytotoxicity against RAW264.7 and did not cause significant inflammatory responses, indicating the good biocompatibility of the silk fibroin nanoparticles, which were similar to the findings of Mercedes G. Montalbán (Montalbán et al., 2018), Alessandra Mari Bossi (Bossi et al., 2021) et al. Moreover, we investigated the drug-loading properties of SNs, and the results showed that SNs have good drug-loading performance, and the loaded SNs have good antibacterial effects. However, the loading rate and encapsulation rate still have room for further improvement, and it is difficult to achieve precision therapy with unfunctionally modified SNs.

To achieve functions including precise drug delivery and immune escape, nanoparticles often require complex functionalization modifications (Guerrini et al., 2018). As an emerging technology, cell membrane bionanotechnology is considered to have great potential for application because of its advantages of improving the biocompatibility of nanoparticles, prolonging the retention time in blood circulation, achieving immune escape and homologous targeting (Zou et al., 2020; Oroojalian et al., 2021). Recent studies have shown that immune cell membrane-mimetic nanoparticles can well target the inflamed endothelium due to the expression of adhesion receptors and that antigenic membrane proteins facilitate their diffusion at the site of inflammation (Park et al., 2021). For example, macrophage membrane-mimetic nanoparticles have been shown to diffuse to sites of systemic inflammation and are used in the management of sepsis (Thamphiwatana et al., 2017); Neutrophil membrane-encapsulated nanoparticles are well aggregated in inflamed osteoarthritic sites and show better tissue penetration than erythrocyte membrane-mimetic particles (Zhang Q. et al., 2018). Moreover, genetic engineering techniques have been innovatively applied to enhance cell membrane functions, greatly increasing the potential of cell membrane bionanotechnology applications. Genetic engineering is a versatile platform to achieve the expression of specific target ligands on cell membranes without disturbing membrane proteins and with significantly lower technical difficulty than chemical modifications (Ai et al., 2020; Park et al., 2021). In this study, we genetically engineered TLR4-expressing RAW264.7 cells and extracted functionalized cell membranes to physically extrude with drug-loaded SNs to successfully prepare MSNCs. Scanning electron microscopy results showed that the surface of MSNCs was not smooth and differed significantly from the smooth surface of SNs. The TEM results demonstrated the distinct shell-core structure of MSNCs, which showed a high-density image of drug-loaded SNs in the middle and a lower-density cell membrane image closely surrounding the outer layer. The DLS results showed that the particle size and zeta potential of MSNCs became larger compared to SNs, indicating the successful coating of CMs, and the change of the surface charge of the particles after coating. The Bradford staining results showed that the cell membrane surface proteins were well preserved during the extraction of cell membranes as well as the preparation of bionanoparticles, indicating that MSNCs could inherit the relevant functions of the source cell surface proteins. What’s more, the western blot results showed that the surface of MSNCs still contained a large number of TLR4, further proving the successful preparation of MSNCs. it can be seen that the drug release rate increased continuously with the increasing time, it indicates that more drug was released; and when *E. coli* combined with MSNCs, the initial release rate of the drug slowed down significantly, but this effect diminished significantly after 8 h. And similar to Gou’s study (Gou et al., 2019), the drug release rate of MSNCs was up to 20% within 48 h in PBS solution with neutral PH. Thus, MSNCs maintained a sustained drug release after binding to bacteria.

We still verified that SNCs have good biocompatibility. We next verified that MSNCs have good antibacterial effects based on their targeted drug-release properties. On the one hand, the functionalized cell membrane on the surface of the nanoparticles could bind specifically to the LPS on bacterial due to TLR4, which made them have significant bacterial binding ability; on the other hand, MSNCs could release antibacterial drugs in a short period of time to kill *E. coli* precisely (Thamphiwatana et al., 2017; Wu et al., 2021). LPS activates TLR4 and leads to the activation and overexpression of MyD88, which in turn activates the NF-κB pathway, leading to NF-κB phosphorylation and translocation from the cytoplasm to the nucleus, promoting the expression of pro-inflammatory cytokines such as IL-1β and IL-6 (Lu et al., 2008; Thamphiwatana et al., 2017; Zhuang et al., 2020). The RT-PCR results showed that the inflammatory gene expression level was significantly increased after LPS stimulation, and the inflammatory gene expression level remained unchanged significantly after treatment with SNs, which further indicated that SNs have good biocompatibility. However, the inflammatory gene expression level was significantly reduced in the SNCs-treated group, suggesting the immunoregulatory ability of SNCs. Based on the properties of cell membranes overexpressing TLR4, we hypothesized that SNCs inhibited inflammatory responses by neutralizing LPS in the microenvironment and effectively reducing the activation of macrophages by LPS. These results suggest that MSNCs possess both precise antibacterial and immunoregulatory properties.

To verify the inhibitory effect of MSNCs on inflammation in periodontal tissues, a LIP mouse model was constructed in this study, and its therapeutic effect was evaluated after the topical administration of treatment. MSNCs have good local storage capacity and tissue penetration and can selectively diffuse to the periodontal inflammation site with good target diffusion of inflammation. The results showed that the level of inflammation in periodontal tissues was significantly lower and tissue destruction was significantly reduced in the MSNCs group compared with the no-treatment and MSNs groups, suggesting that MSNCs effectively reduced inflammation and inhibited bone loss in periodontitis.

## 5 Conclusion

In summary, we constructed an engineered cell membrane-encapsulated nanosystem MSNCs for precise targeting and inhibition of bacteria, and to neutralize LPS in the microenvironment to inhibit inflammatory cell activation. MSNCs effectively inhibited periodontal inflammation and bone loss through dual regulation of antibacterial and immunity. Based on this nanoplatform, genetically engineered modifications with different ligand-receptor patterns can be constructed to respond to immune diseases infected by different pathogens. In addition, this system still has some potential for improvement, such as the drug loading rate can be further optimized and the therapeutic effect can be evaluated over a longer period of time. In conclusion, MSNCs provide a new therapeutic platform for periodontitis, and the dual functional design of antibacterial and immunoregulation provides additional consideration for the treatment of inflammatory diseases with bacterial infections.

## Data Availability

The original contributions presented in the study are included in the article/Supplementary Material, further inquiries can be directed to the corresponding author.

## Supplementary material

Supplementary Figure S1 Drug release profiles of MSNCs and E. coli-treated MSNCs from 0 h to 48 h.

Supplementary Figure S2 Diffusion of MSNCs in vivo. The white curved line indicates the mouse molar; the red arrow and red curved line indicate the main green fluorescence.
