# Supplementary figures and images for: Genetically engineered cell membrane-coated nanoparticles for antibacterial and immunoregulatory dual-function treatment of ligature-induced periodontitis
Source: Front Bioeng Biotechnol. 2023 Jan 25;11:1113367. doi: 10.3389/fbioe.2023.1113367 (PMC9905692; doi:10.3389/fbioe.2023.1113367)

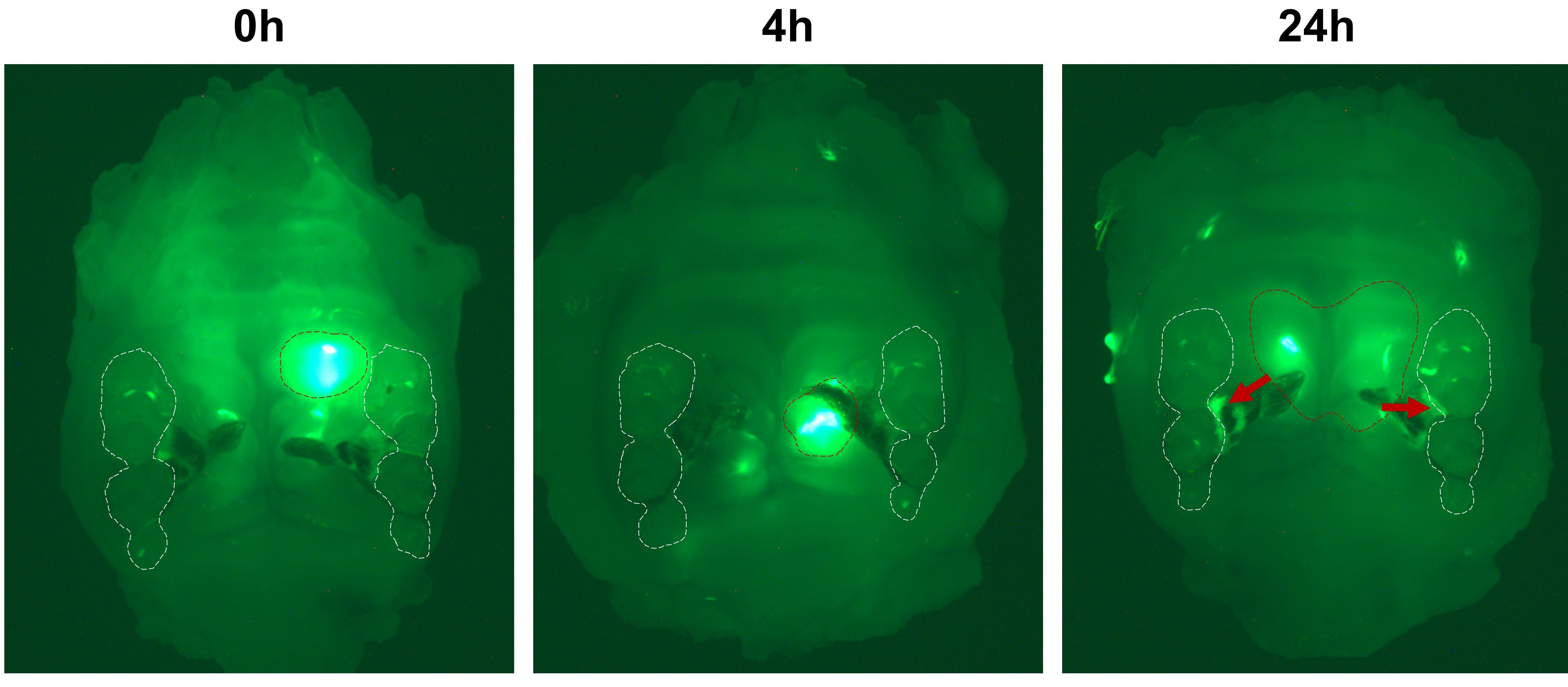

Supplement: Supplementary file 1 [file Image1.JPEG]

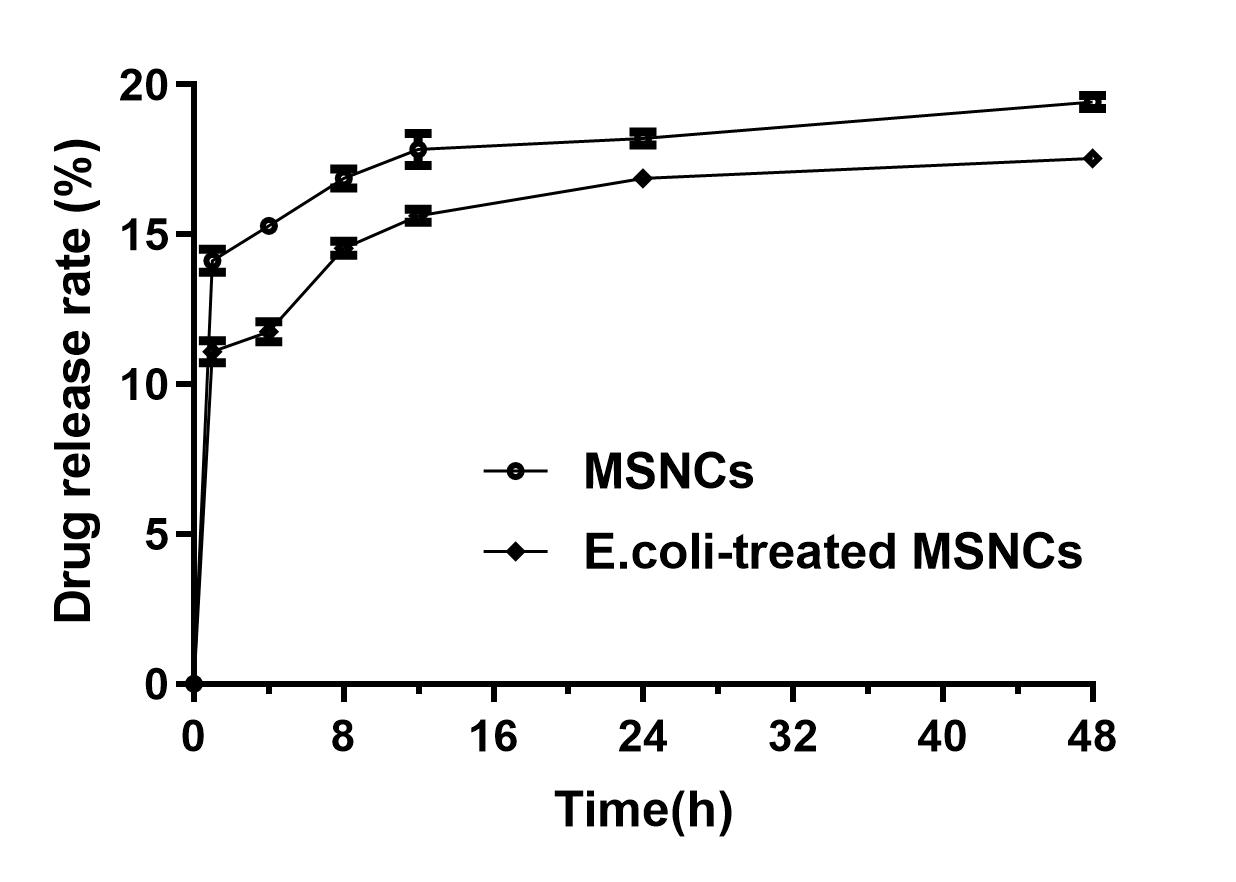

Supplement: Supplementary file 2 [file Image2.JPEG]
